# Comment on “The effect of psychological inflexibility on health-related quality of life, depression, and anxiety in patients with chronic tinnitus without hearing loss”

**DOI:** 10.1590/1806-9282.20231734

**Published:** 2024-05-17

**Authors:** Xiaohong Zhang, Shenggang Yan

**Affiliations:** 1 Haining Hospital of Traditional Chinese Medicine, Department of Otolaryngology – Haining, China.; 2 Haining Cancer Hospital, Haining Cancer Prevention and Treatment Research Institute – Haining, China.

Dear Editor,

We read with great interest the recent study^1^ investigating the relationship between psychological inflexibility and health-related quality of life, depression, and anxiety in individuals coping with chronic tinnitus but without accompanying hearing loss. The exploration of such an aspect of the human experience is both timely and essential, given the increasing prevalence of chronic tinnitus and its often-profound impact on individuals’ overall well-being. The study^1^ analyzes the lesser-explored psychological aspects of chronic tinnitus, highlighting the role of psychological inflexibility in amplifying the difficulties experienced by patients. Through a detailed examination of how psychological inflexibility interacts with health-related quality of life, depression, and anxiety, the researchers offer valuable insights that enhance our understanding of the complex nature of chronic tinnitus. The findings suggest that addressing psychological inflexibility may hold promise as an avenue for intervention and support for individuals grappling with the burdensome effects of chronic tinnitus. This implies that therapeutic approaches aimed at enhancing psychological flexibility could potentially play a crucial role in ameliorating the negative impacts on mental health and overall quality of life for this patient population. Furthermore, the study prompts reflection on the broader implications for healthcare professionals, emphasizing the importance of adopting a holistic and patient-centered approach to the management of chronic conditions such as tinnitus. The integration of mental health considerations alongside traditional medical approaches may prove instrumental in fostering more comprehensive and effective care for these patients. However, we note that the following concerns require further clarification.

First, in reference to Table 1 of the study^1^, the mean value for the control group’s Beck Depression Inventory (BDI) is reported as 5.29, with a corresponding standard deviation of 6.25. Notably, the standard deviation value (6.25) is significantly higher than its corresponding mean (5.29). In statistical terms, when the standard deviation is markedly higher than the mean, it suggests a skewed distribution of data. In such cases, it is advisable to describe the data using the median and interquartile range and to employ the Wilcoxon rank-sum test for between-group comparisons rather than the t-test. Therefore, it is highly recommended to conduct a normality test for continuous data before initiating data analysis. While acknowledging that the ultimate conclusions may not undergo significant changes, employing appropriate statistical methods contributes to the credibility and replicability of the study. By ensuring the robustness of statistical analyses, we can enhance the overall validity of the research findings. We believe that addressing these statistical considerations will strengthen the methodological rigor of the study and contribute to the robustness of its conclusions.

Second, as outlined in the study, the participants comprised individuals with chronic tinnitus. However, the research does not provide an in-depth exploration of the treatment strategies employed for chronic tinnitus, particularly those related to health-related quality of life, depression, and anxiety. A randomized controlled trial^2^ involving 492 tinnitus patients revealed that those receiving specialized treatment exhibited higher health-related quality of life and lower tinnitus severity compared to those receiving usual care. This suggests an association between specialized treatment, improved quality of life, and reduced tinnitus severity. In addition, a study performed by Ibarra-Zarate et al^3^. cautioned against the indiscriminate use of music therapy (MT) as it may exacerbate the condition of tinnitus patients. On the contrary, their results found that for patients with tinnitus who are dealing with stress-related symptoms but not anxiety, it is advisable to consider Binaural Sound Therapy (BST). The evidence presented indicates potential treatment strategies linked to quality of life, stress, and anxiety. However, the current study lacks a detailed account of the treatment strategies employed for individuals with chronic tinnitus.
